# RNA sequencing analysis of monocrotaline-induced PAH reveals dysregulated chemokine and neuroactive ligand receptor pathways

**DOI:** 10.18632/aging.102922

**Published:** 2020-03-16

**Authors:** Genfa Xiao, Tingjun Wang, Wei Zhuang, Chaoyi Ye, Li Luo, Huajun Wang, Guili Lian, Liangdi Xie

**Affiliations:** 1 Department of General Medicine, The First Affiliated Hospital of Fujian Medical University, Fuzhou 350005, People’s Republic of China; 2 Department of Geriatric Medicine, The First Affiliated Hospital of Fujian Medical University, Fuzhou 350005, People’s Republic of China; 3 Fujian Hypertension Research Institute, The First Affiliated Hospital of Fujian Medical University, Fuzhou 350005, People’s Republic of China

**Keywords:** pulmonary arterial hypertension, RNA sequencing, inflammatory/immune response, neuroactive ligand receptor, transcriptional profiling

## Abstract

Pulmonary arterial hypertension (PAH) is a serious disease characterized by elevated pulmonary artery pressure, inflammatory cell infiltration and pulmonary vascular remodeling. However, little is known about the pathogenic mechanisms underlying the disease onset and progression. RNA sequencing (RNA-seq) was used to identify the transcriptional profiling in control and rats injected with monocrotaline (MCT) for 1, 2, 3 and 4 weeks. A total of 23200 transcripts and 280, 1342, 908 and 3155 differentially expressed genes (DEGs) were identified at the end of week 1, 2, 3 and 4, of which Svop was the common top 10 DEGs over the course of PAH progression. Functional enrichment analysis of DEGs showed inflammatory/immune response occurred in the early stage of PAH development. KEGG pathway enrichment analysis of DEGs showed that cytokine-cytokine receptor interaction and neuroactive ligand-receptor interaction were in the initiation and progression of PAH. Further analysis revealed impaired expression of cholinergic receptors, adrenergic receptors including alpha1, beta1 and beta2 receptor, and dysregulated expression of γ-aminobutyric acid receptors. In summary, the dysregulated inflammation/immunity and neuroactive ligand receptor signaling pathways may be involved in the onset and progression of PAH.

## INTRODUCTION

Pulmonary arterial hypertension (PAH) is a life-threatening disease characterized by elevated pulmonary arterial pressure, infiltration of inflammatory cells and pulmonary vascular remodeling, ultimately leading to right heart failure and premature death. People at every age stage may be affected, especially in elderly people over 65 years old, and it is estimated that the prevalence of PAH is about 1% in the global population and increases up to 10% in the elderly [1]. Survival rate of patients with advanced PAH remains quite lower within 5 years [2]. However, little is known about the pathogenic mechanisms underlying the disease onset and progression.

Microarray has been used to detect transcriptional profiling in explanted lungs from various forms of advanced PAH and identified a great deal of information on PAH, including gene expression signatures, potential biomarkers and therapeutic targets [3]. However, microarray has several limitations, including high background level, a limited dynamic detection range and lack of sensitivity in detecting low copy transcripts [4]. High-throughput RNA sequencing (RNA-seq) is a powerful and unbiased tool that allows detection of genome-wide transcriptional profiling. Recently, RNA-seq has emerged as an alternative to microarray, because of its accuracy, sensibility, larger dynamic detection range and higher reproducibility than microarray [4, 5]. Despite these, only fewer studies have used RNA-seq to analyze the PAH transcriptional profiling. The comparison of RNA-seq with microarray in quantifying gene expression level has been carried out in schistosoma-induced pulmonary hypertension, which showed that the correlation between microarray and RNA-seq was lower, especially for low copy transcripts where RNA-seq had a wider dynamic range than microarray [6].

Monocrotaline (MCT) was widely used for the induction of PAH in rats. Our previous study have successfully established a rat PAH model by a single intraperitoneal injection of 40 mg/kg MCT, based on the evidences of significantly elevated mean pulmonary arterial pressure, right ventricular hypertrophy index, and pulmonary artery remodeling indices [7–10]. Microarray studies have provided data on investigation of human end-stage PAH, however, transcriptional profiling of the disease origin was lacking [11]. In the present study, we used the RNA-seq to perform a comprehensive analysis of the transcriptional profiling during the initiation and progression of MCT-induced PAH, aiming to have a better understanding of pathogenic mechanisms underlying the disease onset and progression.

## RESULTS

### Analysis of transcriptome changes in response to MCT treatment

**Figure 1:**
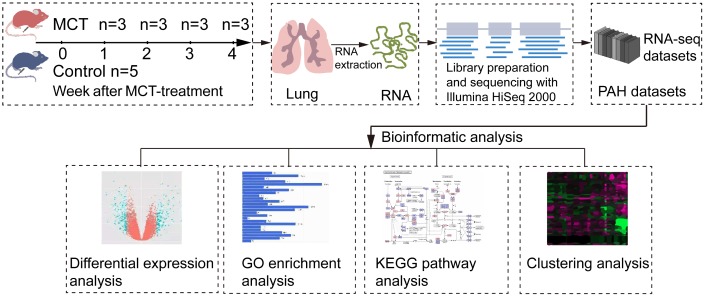
**Schematic workflow of RNA-seq and bioinformatics analysis.** MCT-treatment rats were treated with MCT for 1, 2, 3 and 4 weeks, and then total RNA was isolated from control and MCT-treated rats. After cDNA library preparation and RNA-seq, the datasets were generated and submitted to bioinformatics analysis, including differential expression analysis, GO enrichment analysis, KEGG pathway enrichment analysis and clustering analysis.

To explore the transcriptome changes during the initiation and progression of PAH, we performed RNA-seq analysis of lung tissues isolated from control and MCT-treatment rats that had been treated with MCT for 1, 2, 3 and 4 weeks (Figure 1). RNA-seq generated 54496350.71±5424147.74 raw reads. After removing the low quality reads and adapter sequences, we obtained 53966718.24±5389121.99 clean reads, accounting for 98.88%-99.14% raw reads. Quality control analysis showed the values of Q20 and Q30 were more than 97.85% and 94.61%, respectively (Supplementary Table 1). Then, we mapped the clean reads to rat reference genome using STAR software for each sample, a total of 52905058.24±5343368.73 clean reads was aligned, yielding an average successful mapping rate of 98.02% (Supplementary Table 2).

After assembling and calculation of the transcripts by using FPKM, a total of 23200 transcripts were detected in assembled transcripts, of which 21400, 20575, 20554, 20958 and 20555 transcripts were identified as expression in control, MCT-treatment 1 week, MCT-treatment 2 weeks, MCT-treatment 3 weeks and MCT-treatment 4 weeks, respectively, accounted for 92.24%, 88.68%, 88.59%, 90.34% and 88.59% of expressed transcripts (Table 1). These results indicated that the percentages of expressed transcripts were decreased in the progression of MCT-induced PAH. For those identified transcripts, they could be further divided into 8 intervals, including 0≤FPKM<1, 1≤FPKM<5, 5≤FPKM<10, 10≤FPKM<20, 20≤FPKM<30, 30≤FPKM<40, 40≤FPKM<50 and 50≤FPKM. The majority of the transcripts were in the range of 0≤FPKM<1, accounted for 37.31%-42.69% of the total expressed transcripts. Then, it was the range of 1≤FPKM<5, accounted for 17.21%-19.26% (Supplementary Table 3). As a result, these data indicated that abundances of most transcripts were low or extremely low, which was reported to be difficult for microarray to quantify [4].

**Table 1:** Summary table of expressed transcripts and their percentages.

**Group**	**Expressed transcripts**	**Expressed transcripts%**
CTW	21400	92.24%
MCTW1	20575	88.68%
MCTW2	20554	88.59%
MCTW3	20958	90.34%
MCTW4	20555	88.59%

CTW, control; MCTW1, MCT treatment for 1 week; MCTW2, MCT treatment for 2 weeks; MCTW3, MCT treatment for 3 weeks and MCTW4, MCT treatment for 4 weeks.

### Identification of DEGs induced by MCT

**Figure 2:**
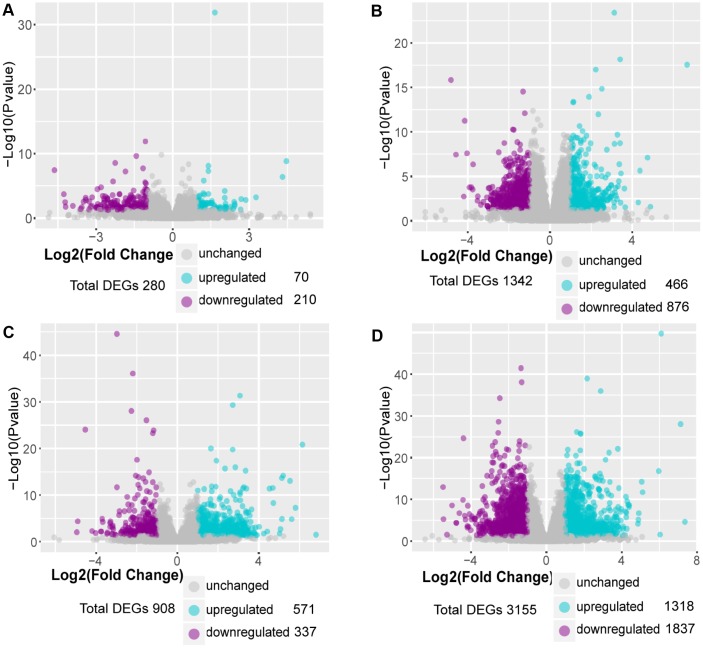
**Identification of DEGs in MCT-induced PAH.** (**A**) DEGs in comparison of MCT-treatment 1 week with control, (**B**) DEGs in comparison of MCT-treatment 2 weeks with control, (**C**) DEGs in comparison of MCT-treatment 3 weeks with control and (**D**) DEGs in comparison of MCT-treatment 4 weeks with control. The DEGs between MCT treatment and control were identified by DESeq2 package in R 3.5.3.

Differentially expressed genes (DEGs) were identified by using a threshold of fold change ≥ 2 and p≤0.05. Differential expression analysis showed that 280 genes were differentially expressed at week 1 compared with control, including 70 upregulated genes and 210 downregulated genes. Using the same threshold, 1342 DEGs were identified at week 2, including 466 upregulated genes and 876 downregulated genes; 908 DEGs were identified at week 3, including 571 upregulated genes and 337 downregulated genes; 3155 DEGs were identified at week 4, including 1318 upregulated genes and 1837 downregulated genes (Figure 2A–2D). These results suggested that the number of upregulated DEGs was gradually increased in the progression of PAH and most of the DEGs were downregulated.

### Identification of common genes in top 10 DEGs induced by MCT

**Figure 3:**
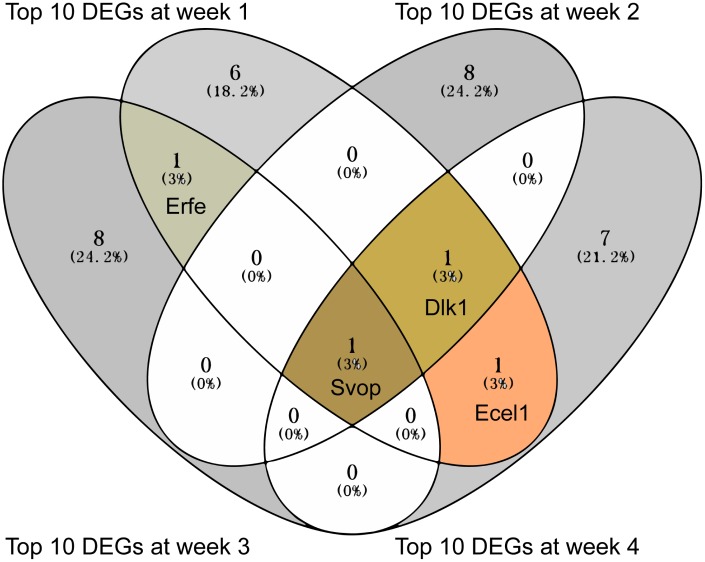
**The overlapped top 10 DEGs showed in Venn diagram.** The overlapped top 10 DEGs were analyzed by Venny 2.1.

The top 10 DEGs with the strongest differential expression between MCT treatment and control were extracted for each differential expression analysis (Supplementary Tables 4–7). Further analysis of the top 10 DEGs using Venny 2.1 showed that Svop was overlapped at the all 4 weeks, Dlk1 was overlapped at week 2, week 3 and week 4 and Ecel1 was overlapped at week 1 and week 4 (Figure 3). Of note, Svop was located in the synaptic vesicle annotated by Gene Ontology (GO) (Table 2).

**Table 2:** The GO annotation of the overlapped top 10 DEGs.

**Gene symbol**	**Biological process**	**Cellular component**	**Molecular function**
Svop	transmembrane transport	synaptic vesicle	transmembrane transporter activity
Ecel1	neuropeptide signaling pathway	component of membrane	endopeptidase activity
Erfe	iron ion homeostasis	extracellular space	receptor binding
Dlk1	negative regulation of notch signaling pathway	component of membrane	calcium ion binding

### GO enrichment analysis of DEGs in MCT-induced PAH

**Figure 4:**
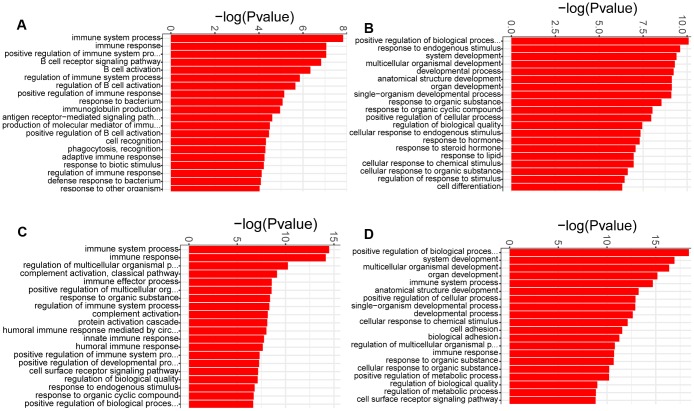
**GO analysis of the DEGs identified in comparison of MCT-treatment with control.** (**A**) biological process terms enriched in DEGs at week 1, (**B**) biological process terms enriched in DEGs at week 2, (**C**) biological process terms enriched in DEGs at week 3 and (**D**) biological process terms enriched in DEGs at week 4. Only the top 20 biological process terms of GO enrichment analysis were showed.

GO enrichment analysis was used to determine the biological process terms of the DEGs at each week. It was showed that almost all the biological process terms were linked to inflammation/immunity at week 1, such as immune system process, immune response, and regulation of immune system process (Figure 4A), demonstrating a role of inflamatory/immune response at the early time point of PAH development. Of note, humoral immunity may play an important role in the early development of PAH, due to enrichment of lots of GO terms including B cell receptor signaling pathway, B cell activation, regulation of B cell activation, antigen receptor−mediated signaling pathway, immunoglobulin production and positive regulation of B cell activation (Figure 4A). Moreover, immune system process and immune response were also the most significantly enriched biological process terms at week 3. In addition to humoral immune response, the complement activation was also significantly enrihced at week 3 (Figure 4C). In contrast, we noticed that the most significantly enriched biological process term of the DEGs at week 2 was positive regulation of biological process. Additionally, a large number of biological process terms associated with development, including system development, developmental process, anatomical structure development and organ development, were significantly enriched (Figure 4B). These similar results were identified at week 4 (Figure 4D). Thus, it was possible that pulmonary vascular remodeling appeared at week 2 and exacerbated at week 4.

### KEGG pathway analysis of DEGs in MCT-induced PAH

**Figure 5:**
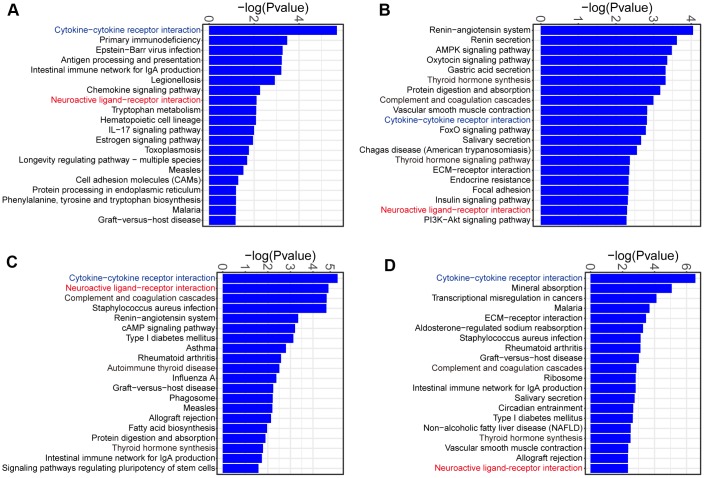
**KEGG pathway analysis of the DEGs identified in comparison of MCT-treatment with control.** (**A**) KEGG pathway terms enriched in DEGs at week 1, (**B**) KEGG pathway terms enriched in DEGs at week 2, (**C**) KEGG pathway terms enriched in DEGs at week 3 and (**D**) KEGG pathway terms enriched in DEGs at week 4. Only the top 20 KEGG pathway terms were showed.

To determine the signaling pathways of DEGs at each week, we used KOBAS to identify KEGG pathways enriched by the DEGs. The majority of the enriched KEGG pathways were related to inflammation/immunity at week 1, such as cytokine-cytokine receptor interaction, chemokine signaling pathway and IL-17 signaling pathway (Figure 5A). Therefore, these results confirmed a role of inflammatory and immune response at the early time point of PAH development. Interestingly, we noticed that DEGs were commonly enriched in cytokine-cytokine receptor interaction and neuroactive ligand-receptor interaction during the onset and progression of PAH (Figure 5A–5D).

### The change of cytokine-cytokine receptor interaction in response to MCT treatment

**Figure 6:**
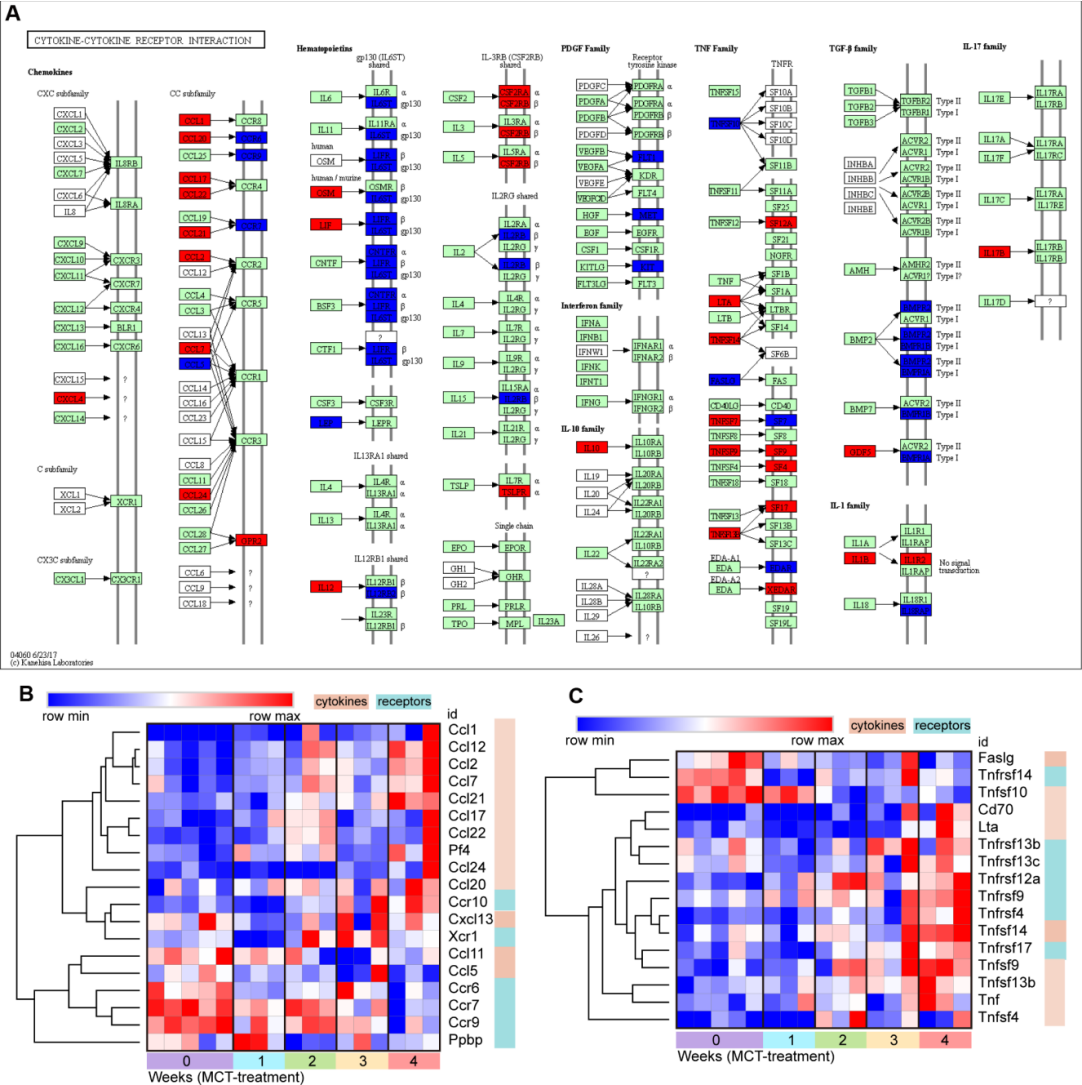
**The change of cytokine-cytokine receptor interaction in response to MCT treatment.** (**A**) KEGG pathway map showing change of cytokine-cytokine receptor interaction, DEGs with relatively increased and reduced expression were shown in red and blue, respectively, while green represented background genes. KEGG pathway only at week 4 was showed. (**B**) Heatmap and hierarchical clustering analysis of DEGs that were annotated in the chemokines and receptors in cytokine-cytokine receptor interaction, (**C**) Heatmap and hierarchical clustering of DEGs that were annotated in the TNF families and receptors in cytokine-cytokine receptor interaction.

The CXC and CC of chemokine subfamilies and TNF families in cytokine-cytokine receptor interaction were further analysis. Hierarchical clustering of DEGs annotated in the CXC and CC subfamilies showed the increase in expression of numerous chemokines, such as Ccl1, Ccl2, Ccl7, Ccl12, Ccl17, Ccl20, Ccl21, Ccl22, Ccl24, Cxcl13 and Cxcl4. However, except Ccr10 and Xcr1, the majority of chemokine receptor expression were reduced, including Ccr6, Ccr7 and Ccr9, reflecting the restricted chemokine receptor signaling (Figure 6A, 6B). Similarly, a fairly large number of TNF ligand families were gradually upregulated, including Tnfsf4, Tnfsf13b, TnfSF14, Tnfsf9, Cd70 and Lta. In contrast to downregulation of chemokine receptors, the expression of TNF ligand family receptors, including Tnfrsf17, Tnfrsf4, Tnfrsf9, Tnfrsf12a, Tnfrsf13c and Tnfrsf13b, were upregulated (Figure 6A, 6C).

### The change of neuroactive ligand-receptor interaction in response to MCT treatment

**Figure 7:**
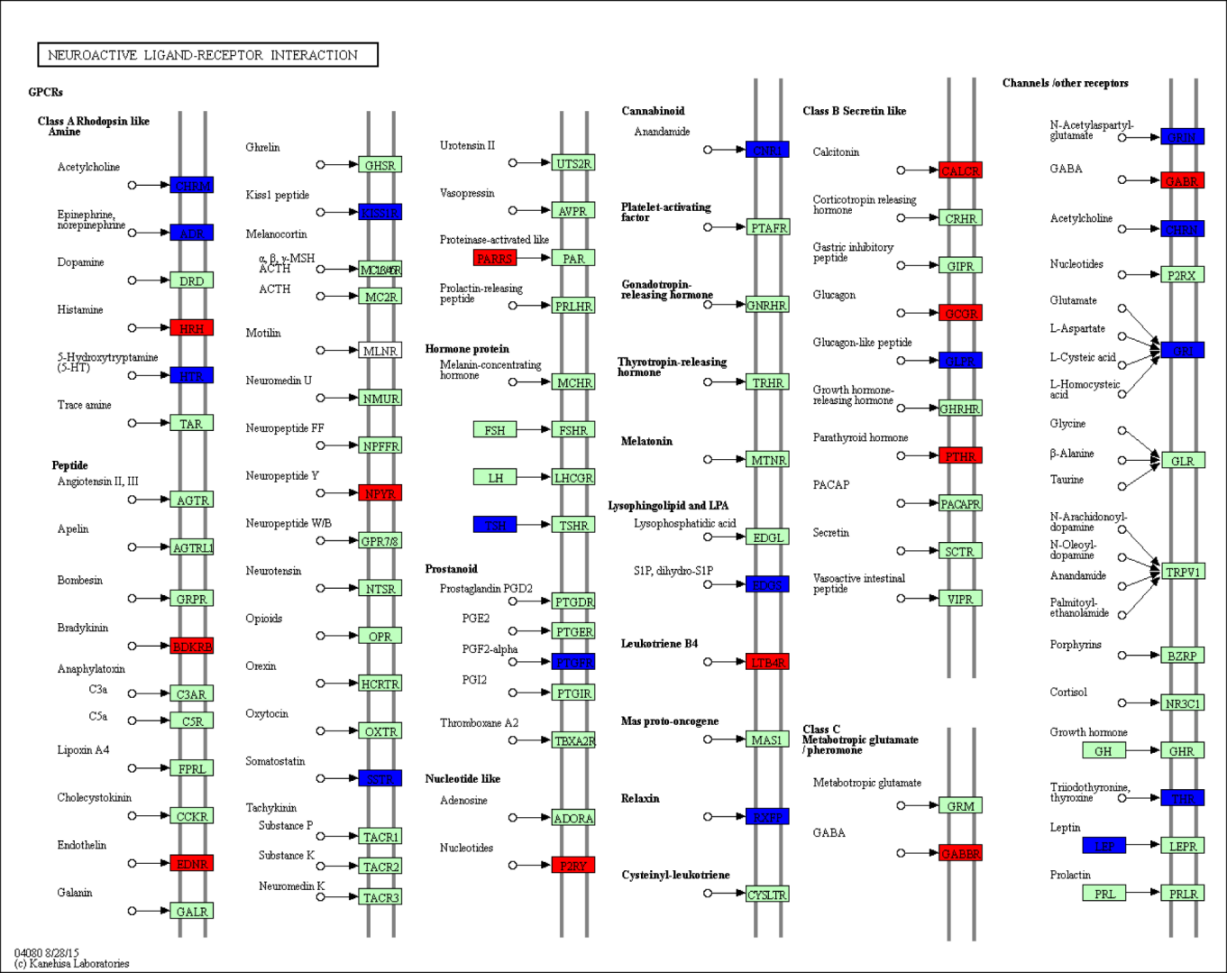
**KEGG pathway map showing change of neuroactive ligand-receptor interaction.** DEGs with relatively increased and reduced expression were shown in red and blue, respectively, while green represented background genes. KEGG pathway only at week 4 was showed.

A series of neuroactive ligand receptors were differentially expressed in neuroactive ligand-receptor interaction pathway, such as downregulated receptors including acetylcholine receptor, epinephrine and norepinephrine receptors, 5-hydroxytryptamine receptor, somatostatin receptor, anandamide receptor, glutamate receptor and glucagon-like peptide receptor, as well as upregulated receptors including γ-aminobutyric acid (GABA) receptor, nucleotides receptor, endothelin receptor, glucagon receptor, neuropeptide Y receptor and bradykinin receptor (Figure 7).

**Figure 8:**
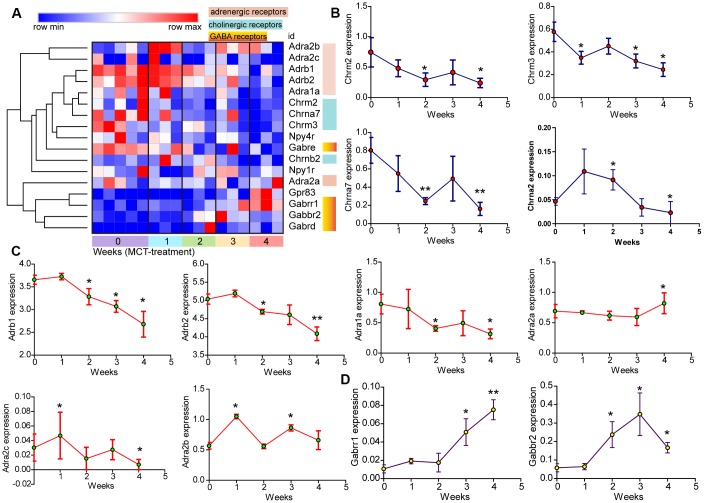
**The change of neuroactive ligand-receptors in MCT-induced PAH.** (**A**) Hierarchical clustering of DEGs that were annotated in the cholinergic and adrenergic receptors as well as GABA receptors. (**B**–**D**) Temporal expression levels of DEGs annotated in neuroactive ligand-receptor interaction. Data were shown as mean ± S.E.M and analyzed by one-way ANOVA followed by Dunnett’s test, n = 3 MCT-treated rats at each week, and n = 5 control. *p<0.05 *vs* control and **p<0.01 *vs* control.

Further hierarchical clustering analysis of cholinergic and adrenergic receptors using Morpheus showed downregulation of muscarinic acetylcholine receptors Chrm2 and Chrm3, as well as nicotinic acetylcholine receptors Chrna7 and Chrnb2 (Figure 8A, 8B). The expression of adrenergic alpha1 and beta receptors, including Adra1a, Adrb1 and Adrb2, were also reduced. By contrast, adrenergic alpha2 receptor expression varied with subtypes: Adra2c expression was not sustained reduced over time, whereas Adra2a and Adra2b expression were elevated (Figure 8A, 8C). These results indicated the dysregulated expression of both sympathetic and parasympathetic receptors in the progression of PAH. In addition, hierarchical clustering analysis also revealed increased expression of GABA receptors Gabbr2 and Gabrr1 (Figure 8A, 8D).

## DISCUSSION

PAH is a substantial global health issue and increasingly diagnosed in the elderly [1]. In the present study, RNA-seq was used to identify transcriptional profiling change during the initiation and progression of MCT-induced PAH. RNA-seq analysis revealed reduced expression of transcripts in the progression of PAH, whereas the number of upregulated DEGs was gradually increased. Functional enrichment analysis of DEGs showed inflammatory/immune response occurred at the early time point of PAH development. In addition, KEGG pathway enrichment analysis of DEGs revealed dysregulated cytokine-cytokine receptor interaction and neuroactive ligand-receptor interaction in the onset and progression of PAH.

Marked perivascular inflammation was present in the lung of PAH and correlated with pulmonary vascular remodeling [12]. The infiltration of macrophage was identified in our previous study [9]. In addition to macrophage, B lymphocyte and its mediated humoral immune response may also play a role in the pathogenesis of PAH, this is due to enrichment of lots of GO terms linked to B cell activation and humoral immunity in this study. The development and progression of PAH were associated with the dysregulated expression of several chemokines and chemokine receptors in the pulmonary vasculature [13]. Interestingly, cytokine-cytokine receptor interaction was persistently enriched in the development and progression of MCT-induced PAH. Further hierarchical clustering analysis revealed dysregulated expression of several chemokines and chemokine receptors. Of these chemokines and chemokine receptors, Ccl2, Ccl7, Ccl20, Ccl21, Cxcl13, Cxcl4, Ccr6 and Ccr7 have already been demonstrated to be associated with pulmonary hypertension [13]. Notably, the expression of several chemokine receptors, such as Ccr6, Ccr7 and Ccr9, were reduced rather than increased in the progression of PAH. It was possible that presence of negative feedback mechanism that restricted the overactivated or prolonged chemokine receptor signaling.

TNFα transgenic mice developed spontaneous PAH and TNFα drove the progression of PAH by suppressing BMPR2 expression and changing NOTCH signaling [14, 15]. Hierarchical clustering analysis also revealed upregulation of the other TNF ligand families and the receptors that were previously less well-appreciated in PAH. Thus, these results may expand the driving role of TNFα families in the pathogenesis of PAH.

In this study, Svop, Ecel1, Dlk1 and Erfe were identified as the overlapped top 10 DEGs, however their roles in PAH were not characterized. Svop was the only overlapped top 10 genes occurred during the initiation and progression of PAH. Previous studies have demonstrated that Svop was an evolutionarily conserved synaptic vesicle protein and localized to neurotransmitter-containing vesicles, additionally, Svop expression was decreased with aging [16, 17]. Ecel1 was found to be preferentially expressed in the central nervous system and sympathetic ganglia [18]. Ecel1 expression was dramatically increased, as motor and sensory nerves were injured [19]. Dlk1 was a somato-dendritic protein expressed in hypothalamic arginine-vasopressin and oxytocin neurons and involved in neuroendocrine [20]. Collectively, it could be inferred that peripheral nervous system was dysfunctional in the PAH lung. Indeed, KEGG pathway analysis of DEGs in MCT-induced PAH revealed that neuroactive ligand-receptor interaction was persistently enriched during the initiation and progression of PAH. The neuroactive ligand-receptor interaction has already been enriched by the previous studies, but no further work was done [21, 22]. A number of neuroactive ligand receptors were identified as differential expression in the present study, of which cholinergic receptor, neuropeptide Y receptor and glutamate receptor have already been known to be associated with the development of pulmonary hypertension [23–25].

The parasympathetic nervous activity was mediated by 2 types of cholinergic receptors, the nicotinic receptor and the muscarinic receptor. Our results showed reduced expression of both nicotinic and muscarinic receptors. The impaired parasympathetic activity was reported in patients with PAH [26]. Hence, the identification of reduced expression of nicotinic and muscarinic receptors may provide an alternative explanation for impaired parasympathetic activity.

Previously, ^123^Iodine-metaiodobenzylguanidine nuclear imaging was applied to evaluate sympathetic innervation of the ventricle in patients with PAH, the marked reduction in heart-to-mediastinum ratio was observed, suggesting an impaired cardiac sympathetic nervous system [27–29]. Similarly, we identified reduced expression of multiple adrenergic receptors in the disease lung, including adrenergic alpha1, beta1 and beta2 receptors. In contrast, the expression of alpha2 adrenergic receptors, alpha2A and alpha2B, were increased. Alpha2 adrenergic receptors played a role in suppressing neurotransmitter release from sympathetic nerves, the elevated expression of Alpha2 adrenergic receptors may suggest the impaired activation of sympathetic nerves in the PAH lung. GABA is the major inhibitory neurotransmitter in the central and peripheral nervous system where it acts at GABA receptors. The identification of elevated GABA receptors, Gabbr2 and Gabrr1, provided the other evidences of inhibited neurotransmitter release from sympathetic nerves. Collectively, our results indicated both impaired sympathetic and parasympathetic receptors in MCT-induced PAH, which were consistent with clinical observation describing decreased chronotropic response to exercise and heart rate recovery in patients with PAH [30].

The increased sympathetic nerve activity has been reported in PAH [31]. It remained unclear whether reduced adrenergic beta receptors themselves were response to chronic sympathetic hyperactivation just as suggested by the previous study [27], this was due to a lack of consistent beneficial effect of beta-adrenergic receptor blockers in patients with PAH [32]. Furthermore, the current pulmonary hypertension guideline advised against the use of beta-adrenergic receptor blockers in PAH patients [33]. The mechanisms underlying reduced expression of sympathetic and parasympathetic receptors remained elusive. It was likely that inflammation was a contributor, since neuroinflammation was identified in MCT-induced PAH [34, 35].

In summary, inflammatory/immune response was identified at the early time point of PAH development. The dysregulated chemokine and neuroactive ligand receptor signaling may be involved in the onset and progression of PAH, thus, providing novel insights into the pathogenic mechanisms of PAH.

## MATERIALS AND METHODS

### Animal and treatment

Sprague-Dawley rats (SD rats), 200-250g, were purchased from Shanghai SLACCAS Laboratory Animal Co., Ltd. (Certificate No. SCXK 2012–0002). The SD rats were raised in the animal room and given food and water *ad libitum*. The PAH model in rats was established by a single intraperitoneal injection of 40 mg/kg MCT (Sigma-Aldrich, CA, USA) as described previously [7–10]. A total of 17 rats were used in this study, 12 rats were randomly assigned into 4 groups and treated with MCT (n = 3, each MCT-treatment group). To increase the statistical power, 5 remaining rats were served as control and treated with saline. The MCT-treated rats were sacrificed at the end of week 1, 2, 3 and 4, and control rats were sacrificed at the time when MCT was given (week 0), and each time when monocrotaline-treated rats were sacrificed, at the end of week 1, 2, 3 and 4. The lungs were immediately isolated and frozen in the liquid nitrogen and then stored at -80°C. All efforts were made to diminish suffering by using sodium pentobarbital anesthesia. The procedures have been conducted in accordance with the ethical standards and were approved by the Laboratory Animal Welfare and Ethics Committee of Fujian Medical University (Approval No. 2017–070, Fuzhou, China).

### RNA extraction

Total RNA was extracted from 50 mg lung tissues using 1 mL Trizol reagent (Life Technology, USA) following manufacturer’s instructions, as previously described [36]. RNA integrity and quality were assessed by gel electrophoresis. The RNA concentration and purity were determined at A260 nm and A280 nm wavelengths using NanoDrop^TM^ instruments (Thermo Scientific, USA), total RNA with high quality was used for cDNA library preparation.

### cDNA library preparation and RNA-seq

cDNA Library preparation and RNA-seq were performed on an Illumina HiSeq 2000 platform by Genenergy Biotechnology (Shanghai) Co., Ltd.

### Bioinformatics analysis

Bioinformatic analysis tools, including FastQC, STAR, StringTie, Cufflinks-Cuffmerge, DESeq2, TopGO, KOBAS, Venny 2.1 and Morpheus were used in this study. Briefly, the quality of generated reads was assessed by FastQC software (http://www.bioinformatics.babraham.ac.uk/projects/fastqc/). The alignment of clean reads to rat reference genome was performed using STAR software (https://github.com/alexdobin/STAR). The transcripts were assembled by using the StringTie and Cufflinks-Cuffmerge softwares (http://ccb.jhu.edu/software/stringtie/). The values of transcript expression in each sample were determined by calculation of fragments per kilobase of transcript per million fragments mapped (FPKM). Generally, as the FPKM of a transcript was not less than 0.1, the transcript was then regarded as expression. The identification of DEGs between MCT-treatment groups and control was performed by using DESeq2 software with a threshold of fold-change ≥ 2 and p≤0.05. Hierarchical clustering analysis and heatmap creation were performed by Morpheus (https://software.broadinstitute.org/morpheus/). KOBAS 3.0 was used for KEGG pathway enrichment analysis of the DEGs (http://kobas.cbi.pku.edu.cn/). TopGO (http://www.bioconductor.org/packages/release/bioc/html/topGO.html) was used for gene ontology enrichment analysis of the DEGs. The overlapped top 10 DEGs were determined by Venny 2.1 (https://bioinfogp.cnb.csic.es/tools/venny/index.html).

### Statistical analysis

Data are shown as mean ± S.E.M. Multiple comparisons to control were performed with one-way ANOVA followed by Dunnett’s test in R 3.5.3. *P* values of <0.05 were regarded as significant. Further details of the statistical analysis were provided in the figure legends.

## Supplementary Material

Supplementary Tables
