# Supplementary material for: RNA sequencing analysis of monocrotaline-induced PAH reveals dysregulated chemokine and neuroactive ligand receptor pathways
Source: Aging (Albany NY). 2020 Mar 16;12(6):4953–69. doi: 10.18632/aging.102922 (PMC7138548; doi:10.18632/aging.102922)
Supplement: Supplementary Tables [file aging-12-102922-s001..pdf]

SUPPLEMENTARY TABLES

Supplementary Table 1. The summary of raw reads, clean reads and values of Q20 and Q30.

Sample	Raw reads	Clean reads	Average length	Clean reads %	Q20 %	Q30 %
CTW0	54347150	53881330	142.4237	99.14%	98.5%	95.95%
CTW1	55196452	54686818	143.1568	99.08%	98.40%	95.60%
CTW2	47309544	46837854	142.83	99%	98.25%	95.40%
CTW3	48786120	48263046	143.7846	98.93%	98.10%	95%
CTW4	52642514	52102274	143.4323	98.97%	98.15%	95.20%
MCTW11	55426314	54904968	141.5576	99.06%	98.55%	95.95%
MCTW12	57082004	56522476	143.0275	99.02%	98.35%	95.55%
MCTW13	70152968	69475748	141.2986	99.03%	98.45%	95.65%
MCTW21	60554304	59965206	142.1482	99.03%	98.40%	95.65%
MCTW22	57123236	56609990	142.7558	99.10%	98.45%	95.70%
MCTW23	48982862	48461514	141.7249	98.94%	98.30%	95.45%
MCTW31	52163224	51677098	143.162	99.07%	98.45%	95.70%
MCTW32	47903478	47366610	142.7532	98.88%	97.85%	94.61%
MCTW33	56784038	56283860	142.2004	99.12%	98.45%	95.70%
MCTW41	52785414	52223502	142.6143	98.94%	98.25%	95.30%
MCTW42	58353368	57821720	143.4674	99.09%	98.50%	95.85%
MCTW43	50844972	50350196	142.5338	99.03%	98.45%	95.70%

CTW, control; MCTW1, MCT treatment for 1 week; MCTW2, MCT treatment for 2 weeks; MCTW3, MCT treatment for 3 weeks and MCTW4, MCT treatment for 4 weeks. n = 3 for each MCT-treatment group, n = 5 for control.

Supplementary Table 2. Summary table of clean reads and their alignments.

Sample	Total clean reads	Total mapped	Mapped ratio(%)	Unique mapped	Reads Proper pair
CTW0	53881330	52967071	98.30%	49310754	49310624
CTW1	54686818	53691090	98.20%	49282698	49282528
CTW2	46837854	45969162	98.10%	42655997	42655838
CTW3	48263046	47206358	97.80%	43476522	43476334
CTW4	52102274	51111663	98.10%	47765204	47765054
MCTW11	54904968	54001990	98.40%	49431346	49431200
MCTW12	56522476	55501901	98.20%	51635734	51635574
MCTW13	69475748	68294498	98.30%	62718946	62718758
MCTW21	59965206	59006189	98.40%	53633769	53633596
MCTW22	56609990	55557815	98.10%	50691175	50691012
MCTW23	48461514	47552665	98.10%	42742459	42742302
MCTW31	51677098	50669321	98%	46502994	46502810
MCTW32	47366610	46042923	97.20%	42934658	42934372
MCTW33	56283860	54948575	97.60%	49911096	49910906
MCTW41	52223502	51259212	98.20%	47032044	47031836
MCTW42	57821720	56286553	97.30%	51767767	51767558
MCTW43	50350196	49319004	98%	45371920	45371782

CTW, control; MCTW1, MCT treatment for 1 week; MCTW2, MCT treatment for 2 weeks; MCTW3, MCT treatment for 3 weeks and MCTW4, MCT treatment for 4 weeks. n = 3 for each MCT-treatment group, n = 5 for control.

Supplementary Table 3. The distribution ranges of expressed transcript abundances.

Sample	0≤FPKM<1	1≤FPKM<5	5≤FPKM<10	10≤FPKM<20	20≤FPKM<30	30≤FPKM<40	40≤FPKM<50	50≤FPKM
CTW0	8843(38.12%)	4035(17.39%)	2801(12.07%)	3097(13.35%)	1458(6.28%)	827(3.56%)	439(1.89%)	1700(7.33%)
CTW1	9024(38.9%)	4306(18.56%)	2732(11.78%)	2771(11.94%)	1288(5.55%)	736(3.17%)	514(2.22%)	1829(7.88%)
CTW2	8657(37.31%)	4130(17.8%)	2823(12.17%)	3106(13.39%)	1484(6.4%)	828(3.57%)	505(2.18%)	1667(7.19%)
CTW3	8847(38.13%)	4056(17.48%)	2786(12.01%)	2978(12.84%)	1378(5.94%)	803(3.46%)	490(2.11%)	1862(8.03%)
CTW4	8900(38.36%)	4068(17.53%)	2712(11.69%)	3026(13.04%)	1451(6.25%)	822(3.54%)	458(1.97%)	1763(7.6%)
MCTW11	9126(39.34%)	4242(18.28%)	2739(11.81%)	2818(12.15%)	1266(5.46%)	744(3.21%)	484(2.09%)	1781(7.68%)
MCTW12	8936(38.52%)	3993(17.21%)	2764(11.91%)	3040(13.1%)	1447(6.24%)	818(3.53%)	513(2.21%)	1689(7.28%)
MCTW13	9307(40.12%)	4252(18.33%)	2660(11.47%)	2722(11.73%)	1212(5.22%)	741(3.19%)	495(2.13%)	1811(7.81%)
MCTW21	9103(39.24%)	4318(18.61%)	2760(11.9%)	2700(11.64%)	1254(5.41%)	706(3.04%)	525(2.26%)	1834(7.91%)
MCTW22	8886(38.3%)	4182(18.03%)	2775(11.96%)	2879(12.41%)	1360(5.86%)	743(3.2%)	492(2.12%)	1883(8.12%)
MCTW23	9326(40.2%)	4206(18.13%)	2660(11.47%)	2605(11.23%)	1283(5.53%)	715(3.08%)	512(2.21%)	1893(8.16%)
MCTW31	8795(37.91%)	4022(17.34%)	2870(12.37%)	2990(12.89%)	1418(6.11%)	790(3.41%)	506(2.18%)	1809(7.8%)
MCTW32	8699(37.5%)	4117(17.75%)	2897(12.49%)	3119(13.44%)	1491(6.43%)	801(3.45%)	451(1.94%)	1625(7%)
MCTW33	9338(40.25%)	4209(18.14%)	2602(11.22%)	2630(11.34%)	1234(5.32%)	715(3.08%)	475(2.05%)	1997(8.61%)
MCTW41	9904(42.69%)	4344(18.72%)	2368(10.21%)	2401(10.35%)	1165(5.02%)	640(2.76%)	448(1.93%)	1930(8.32%)
MCTW42	9035(38.94%)	4273(18.42%)	2659(11.46%)	2672(11.52%)	1290(5.56%)	750(3.23%)	508(2.19%)	2013(8.68%)
MCTW43	9083(39.15%)	4468(19.26%)	2725(11.75%)	2606(11.23%)	1228(5.29%)	690(2.97%)	453(1.95%)	1947(8.39%)

CTW, control; MCTW1, MCT treatment for 1 week; MCTW2, MCT treatment for 2 weeks; MCTW3, MCT treatment for 3 weeks and MCTW4, MCT treatment for 4 weeks. n = 3 for each MCT-treatment group, n = 5 for control.

Supplementary Table 4. The top 10 DEGs in comparison of MCT-treatment 1 week with control.

Symbol	Gene name	log2FoldChange	Pvalue
Pate4	prostate and testis expressed 4	3.273006	0.000557113
Krt12	keratin 12	2.868081	0.001534974
Svop	SV2 related protein	2.689407	0.001093115
Cxcl6	C-X-C motif chemokine ligand 6	2.046318	0.000707697
Erfe	erythroferrone	2.042368	5.75047E-05
Aass	Aminoadipate-Semialdehyde Synthase	-2.96848	0.018236318
Tgm6	transglutaminase 6	-3.15424	0.000570532
Tnni1	troponin I1, slow skeletal type	-3.27371	0.002540156
Erp27	endoplasmic reticulum protein 27	-4.21826	0.003198764
Lair1	leukocyte-associated immunoglobulin-like receptor 1	-4.63501	3.62254E-08

Supplementary Table 5. The top 10 DEGs in comparison of MCT-treatment 2 weeks with control.

Symbol	Gene Name	log2FoldChange	Pvalue
Svop	SV2 related protein	6.664369	2.93E-18
Gdf15	growth differentiation factor 15	3.412863	7.27E-19
Mmp12	matrix metallopeptidase 12	3.407787	1.87E-09
Erfe	erythroferrone	3.274235	2.06E-10
Ecel1	endothelin converting enzyme-like 1	3.128768	2.24E-09
Hcn3	hyperpolarization-activated cyclic nucleotide-gated potassium channel 3	-3.74328	4.49E-07
Dlk1	delta like non-canonical Notch ligand 1	-3.82786	0.000169
Sun5	Sad1 and UNC84 domain containing 5	-4.03929	2.63E-08
Cacng3	calcium voltage-gated channel auxiliary subunit gamma 3	-4.57397	3.69E-08
Prss29	protease, serine, 29	-4.81529	1.49E-16

Supplementary Table 6. The top 10 DEGs in comparison of MCT-treatment 3 weeks with control.

Symbol	Gene Name	log2FoldChange	Pvalue
Svop	SV2 related protein	6.142864	1.51E-21
Ano3	anoctamin 3	5.540536	9.41E-14
Lctl	lactase-like(Lctl)	5.196691	6.53E-15
Cd209a	CD209a molecule	5.140403	2.31E-14
Gdf3	growth differentiation factor 3	4.685729	2.93E-12
Amelx	amelogenin, X-linked	-3.17676	0.000346
Esrrb	estrogen-related receptor beta	-3.29573	4.56E-05
Adprhl1	ADP-ribosylhydrolase like 1	-3.67701	5.79E-05
Dlk1	delta like non-canonical Notch ligand 1	-4.53374	9.47E-25
Klk6	kallikrein related-peptidase 6	-4.89908	4.31E-05

Supplementary Table 7. The top 10 DEGs in comparison of MCT-treatment 4 weeks with control.

Symbol	Gene Name	log2FoldChange	Pvalue
Ecel1	endothelin converting enzyme-like 1	6.091016	1.89E-50
Svop	SV2 related protein	5.96079	1.72E-17
Mcpt1l1	mast cell protease 1-like 1	5.109331	2E-12
Cma1	chymase 1	5.063219	6.08E-15
Spp1	secreted phosphoprotein 1	4.218197	2.45E-11
Dlk1	delta like non-canonical Notch ligand 1	-4.09894	3.49E-07
Vipr1	Vasoactive Intestinal Peptide Receptor 1	-4.40789	2.09E-25
Asb15	ankyrin repeat and SOCS box containing 15	-4.43624	1.38E-10
Impg1	interphotoreceptor matrix proteoglycan 1	-4.62783	1.92E-07
Opcml	opioid binding protein/cell adhesion molecule-like	-5.00968	2.86E-09
